# Systematic Ocular Phenotyping of Knockout Mouse Lines Identifies Genes Associated With Age-Related Corneal Dystrophies

**DOI:** 10.1167/iovs.66.5.7

**Published:** 2025-05-05

**Authors:** Andrew Briere, Peter Vo, Benjamin Yang, David Adams, Takanori Amano, Oana Amarie, Zorana Berberovic, Lynette Bower, Steve D. M. Brown, Samantha Burrill, Soo Young Cho, Sharon Clementson-Mobbs, Abigail D'souza, Mohammad Eskandarian, Ann M. Flenniken, Helmut Fuchs, Valerie Gailus-Durner, Yann Hérault, Martin Hrabe de Angelis, Shundan Jin, Russell Joynson, Yeon Kyung Kang, Haerim Kim, Hiroshi Masuya, Hamid Meziane, Ki-Hoan Nam, Hyuna Noh, Lauryl M. J. Nutter, Marcela Palkova, Jan Prochazka, Miles Joseph Raishbrook, Fabrice Riet, Jason Salazar, Radislav Sedlacek, Mohammed Selloum, Kyoung Yul Seo, Je Kyung Seong, Hae-Sol Shin, Toshihiko Shiroishi, Michelle Stewart, Karen Svenson, Masaru Tamura, Heather Tolentino, Sara Wells, Wolfgang Wurst, Atsushi Yoshiki, Louise Lanoue, K. C. Kent Lloyd, Brian C. Leonard, Michel J. Roux, Colin McKerlie, Ala Moshiri, for The International Mouse Phenotyping Consortium

**Affiliations:** 1 Touro University California College of Osteopathic Medicine, Vallejo, California, United States; 2 California Northstate University College of Medicine, Elk Grove, California, United States; 3 University of California Davis School of Medicine, Sacramento, California, United States; 4 The Wellcome Trust Sanger Institute, Wellcome Genome Campus, Hinxton, Cambridge, United Kingdom; 5 RIKEN BioResource Research Center, Tsukuba, Japan; 6 Institute of Experimental Genetics, German Mouse Clinic, Helmholtz Zentrum München, Neuherberg, Germany; 7 The Centre for Phenogenomics, Lunenfeld-Tanenbaum Research Institute, Mount Sinai Hospital, Toronto, Ontario, Canada; 8 Mouse Biology Program, University of California Davis, Davis, California, United States; 9 Mary Lyon Centre, Medical Research Council, Harwell Institute, Harwell, United Kingdom; 10 The Jackson Laboratory, Bar Harbor, Maine, United States; 11 Department of Molecular and Life Science, Hanyang University, Seoul, Republic of Korea; 12 Université de Strasbourg, CNRS UMR 7104, INSERM U 1258, IGBMC, Institut Clinique de la Souris, PHENOMIN, Illkirch-Graffenstaden, France; 13 Chair of Experimental Genetics, TUM School of Life Sciences, Technische Universität München, Freising, Germany; 14 German Center for Diabetes Research (DZD), Neuherberg, Germany; 15 College of Veterinary Medicine, Seoul National University, Seoul, Republic of Korea; 16 Laboratory Animal Center, Korea Research Institute of Bioscience and Biotechnology, Daejeon, Republic of Korea; 17 The Centre for Phenogenomics, The Hospital for Sick Children, Toronto, Ontario, Canada; 18 Czech Centre for Phenogenomics, Institute of Molecular Genetics of the Czech Academy of Sciences, 252 50 Vestec, Czech Republic; 19 Department of Ophthalmology, Institute of Vision Research, Yonsei University College of Medicine, Seoul, Republic of Korea; 20 Laboratory of Developmental Biology and Genomics, Research Institute of Veterinary Science, BK21 Plus Program for Advanced Veterinary Science, College of Veterinary Medicine and Interdisciplinary Program for Bioinformatics, Seoul National University, Seoul, Republic of Korea; 21 Institute of Developmental Genetics, Helmholtz Zentrum München, Neuherberg, Germany; 22 Department of Surgery, School of Medicine, University of California Davis, Sacramento, California, United States; 23 Department of Surgical and Radiological Sciences, School of Veterinary Medicine, University of California Davis, Davis, California, United States; 24 Department of Laboratory Medicine & Pathobiology, Faculty of Medicine, University of Toronto, Toronto, Ontario, Canada; 25 Department of Ophthalmology & Vision Science, School of Medicine, University of California Davis, Sacramento, California, United States

**Keywords:** corneal dystrophy, molecular genetics, tears, corneal wound healing, dry eyes

## Abstract

**Purpose:**

This study investigates genes contributing to late-adult corneal dystrophies (LACDs) in aged mice, with potential implications for late-onset corneal dystrophies (CDs) in humans.

**Methods:**

The International Mouse Phenotyping Consortium (IMPC) database, containing data from 8901 knockout mouse lines, was filtered to include late-adult mice (49+ weeks) with significant (*P* < 0.0001) CD phenotypes. Candidate genes were mapped to human orthologs using the Mouse Genome Informatics group, with expression analyzed via PLAE and a literature review for prior CD associations. Comparative analyses of LACD genes from IMPC and established human CD genes from IC3D included protein interactions (STRING), biological processes (PANTHER), and molecular pathways (KEGG).

**Results:**

Analysis identified 14 genes linked to late-adult abnormal corneal phenotypes. Of these, 2 genes were previously associated with CDs in humans, while 12 were novel. Seven of the 14 genes (50%) were expressed in the human cornea based on single-cell transcriptomics. Protein–protein interactions via STRING showed several significant interactions with known human CD genes. PANTHER analysis identified six biological processes shared with established human CD genes. Two genes (*Rgs2* and *Galnt9*) were involved in pathways related to human corneal diseases, including cGMP-PKG signaling, mucin-type O-glycan biosynthesis, and oxytocin signaling. Other candidates were implicated in pathways such as pluripotency of stem cells, MAPK signaling, WNT signaling, actin cytoskeleton regulation, and cellular senescence.

**Conclusions:**

This study identified 14 genes linked to LACD in knockout mice, 12 of which are novel in corneal biology. These genes may serve as potential therapeutic targets for treating corneal diseases in aging human populations.

The human cornea plays a pivotal role in visual acuity and ocular protection.^1^ However, it is subject to a spectrum of disorders known as corneal dysmorphologies (CDs), which describe a collection of multifactorial eye disorders that result in progressive vision loss.^2^ These can be categorized into two broad groups: regressive degeneration corneal dystrophies and corneal dysplasias, driven by defective growth and differentiation. CD may present at birth or develop insidiously during various stages of life, resulting in clinical manifestations that range from subtle to debilitating vision loss, pain, photophobia, or foreign body sensations.^3^

The cornea is composed of five layers, each with a unique function in maintaining its integrity and transparency. These layers include the epithelium, Bowman's layer, the stroma, Descemet's membrane, and the endothelium. CD occurs with degradation or accumulation of material within one or more of these layers.^4^ The IC3D classification system, established by the International Committee for the Classification of Corneal Dystrophies (IC3D), proposes seven major categories of CD: Epithelial and Subepithelial Dystrophies, Bowman Layer Dystrophies, Stromal Dystrophies, Descemet Membrane Dystrophies, Endothelial Dystrophies, Unspecified Stromal Dystrophies, and Miscellaneous Dystrophies.^5^ The IC3D has identified and published CD genes within this classification system.^5^

The use of knockout mice offers a potent strategy for investigating genes associated with CD. The International Mouse Phenotyping Consortium (IMPC) is a global initiative of 21 centers that produces and phenotypes knockout mice for research purposes. These knockout mice undergo a standardized phenotyping pipeline, where a wide range of traits, including ocular phenotypes, are rigorously assessed.^6^

The IMPC utilizes specialized pipelines to assess phenotypes at various developmental stages, encompassing both early-adult and late-adult evaluations. The early-adult pipeline concentrates on the initial phases of mouse development, typically examining mice between 9 to 15 weeks old.^6^ This phase delves into the immediate effects of gene knockouts on traits such as growth, organ development, and behavior manifesting during early life stages. The late-adult pipeline, a subset of the early-adult pipeline, is dedicated to the assessment of phenotypes in mice 49 weeks and older.^6^ It provides insights into the long-term consequences of gene disruptions in mice, shedding light on age-related phenotypic changes, diseases, and characteristics not apparent during earlier stages, with potential implications for age-related CD development in humans.

In recent years, research has increasingly highlighted various networks of signaling pathways and biological processes crucial for ocular health and corneal disease. For instance, the cyclic guanosine monophosphate (cGMP) pathway regulates collagen synthesis in the eye, while mucin glycoproteins, particularly O-glycans, protect the cornea and conjunctiva from physical, chemical, and microbial damage.^7^^,^^8^ Oxytocin is essential for maintaining ocular surface homeostasis, and limbal stem cell regulation is critical for corneal epithelial regeneration.^9^^,^^10^ The MAPK signaling pathway is involved in corneal wound healing and dry eye disease, WNT signaling influences corneal epithelial stratification, regulation of actin cytoskeleton controls tight junction permeability in the cornea, and cellular senescence facilitates the turnover of damaged epithelial cells.^11^^–^^14^ This study aims to discover additional genes relevant to corneal biology in the aging population through an unbiased screening of systematically phenotyped knockout mouse lines.

## Materials and Methods

### Animals and Phenotyping

The IMPC knockout process involves the disruption of protein-coding genes within the mouse genome, followed by rigorous genetic quality control assessment of the mutant mouse lines. Once the genetic quality is confirmed, the consortium generates cohorts of at least seven female and seven male mice for each mutant line. These mice are then subjected to thorough phenotyping, conducted in parallel with age- and sex-matched wild-type (WT) control mice, which are also produced at the same specialized production center.^6^

The IMPC employs two methods to produce their mouse lines, CRISPR/Cas9 editing and embryonic stem cell–derived mice, both on the C57BL/6N strain background.^15^^,^^16^ The phenotypes they identify are systematically described using standardized mammalian phenotyping ontology terms developed by the Mouse Genome Informatics group (MGI).^17^ The zygosity of the mutant lines is also determined at this time, distinguishing between homozygous (HOM), heterozygous (HET), and hemizygous (HEM) conditions.^6^ For further information and to access their comprehensive work, visit the IMPC website at http://www.mousephenotype.org.

This study analyzed Data Release 21.0, which was released on May 7, 2024, and queried on May 10, 2024. In this release, IMPC phenotyped a total of 8901 unique genes, encompassing 9594 mutant lines, and identified 106,561 phenotype hits with a significance level of *P* < 0.0001.^18^

All procedures carried out at IMPC centers comply with strict local, state, and national regulatory guidelines and uphold the principles outlined in the Animal Research: Reporting of In Vivo Experiments guidelines, which aim to standardize and enhance the quality and reproducibility of animal research. Additionally, a Housing and Husbandry protocol is followed, encompassing a set of both mandatory and optional procedures that guide international mouse experimentation.^15^^,^^16^ Furthermore, the consortium ensures that all procedures involving live animals are reviewed and approved by associated institutional animal care and use committees or their equivalent entities, underpinning their commitment to the highest standards of animal welfare.

### Bioinformatics

The exploration of late-adult CD (LACD) phenotypes involved a methodical assessment of genes within the IMPC's online data set to validate the presence of corneal abnormalities. This process entailed querying the IMPC using the term *cornea* in the phenotype search and filtering to focus only on late-adult mice with a significant (*P* < 0.0001) CD phenotype. Focusing on late-adult abnormalities, mouse lines with corneal phenotypes in early adulthood (age <16 weeks) were excluded. Genes linked to LACD phenotypes were manually curated to exclude possible false positives and then subjected to a comprehensive literature review, investigating documented mouse models and their associated corneal phenotypic anomalies in both humans and mice. Additionally, human orthologs of all candidate LACD genes underwent analysis of corneal gene expression and predicted protein-protein interactions and functional pathways, both within the set of candidate genes and against a set of 48 previously established human CD genes, identified from the IC3D.^19^ This analysis was conducted using established bioinformatics tools: Platform for Analysis of Sceiad (PLAE) was used to identify gene expression of human orthologs in the cornea, the Search Tool for the Retrieval of interacting Genes/Proteins (STRING) within the Cytoscape platform (version 3.10.2) was used to analyze protein–protein interactions, Protein Analysis THrough Evolutionary Relationships (PANTHER) was used to compare and contrast biological processes, and Kyoto Encyclopedia of Genes and Genomes (KEGG) within the Database for Annotation, Visualization, and Integrated Discovery website (DAVID) was used to assess whether candidate and established human CD genes were known to be involved in cellular pathways or signaling cascades.^20^^–^^25^

STRING was used to analyze protein–protein interactions within the candidate LACD gene set and protein–protein interactions, including the 48 established human CD genes. STRING allows for the inclusion of a specified number of additional interacting proteins into the specified protein query set. Additional queries were conducted with 10 additional interactor proteins in the analysis within the candidate gene set, as well as between the candidate LACD genes and established human CD genes. Another query was conducted to analyze protein interaction between candidate CD genes and CD genes known to be involved in early-adult CD mice only.^19^ The STRING database supplied confidence scores to facilitate comparative assessments of gene interactions, adhering to the thresholds recommended by the database designers: 0.15 to 0.4 for low confidence, 0.4 to 0.7 for medium confidence, 0.7 to 0.9 for high confidence, and >0.9 for the highest level of confidence in alignment with the study's methodology.^21^ Queries were run at all confidence levels, but only interactions found to be medium or higher were investigated further.

Candidate LACD genes found to be expressed in the human cornea via PLAE were analyzed individually in STRING at the highest confidence level with 10 additional interactor proteins to establish potential functional networks.

PANTHER biological processes for candidate LACD and established human CD genes were assessed separately and then compared. The gene sets were analyzed by entering corresponding STRING IDs of each gene in the gene set with settings, list type: ID list, organism: Homo sapiens, analysis: Functional classification viewed in gene list. Resulting lists were manually inspected and confirmed. Individual biological processes were recorded from the gene list, and bar graphs depicting biological process categories of the full gene set were generated.

KEGG pathways were assessed for the candidate CD gene set and the established CD gene set by manual curation on the KEGG website, generating a comprehensive list of all associated pathways. Both gene sets were then processed through DAVID, generating a list of pathways found to be significant based on the provided inputs. Candidate CD genes, established human CD genes, and additional interactor protein genes were then annotated on significant pathways.

## Results

**Figure 1.**
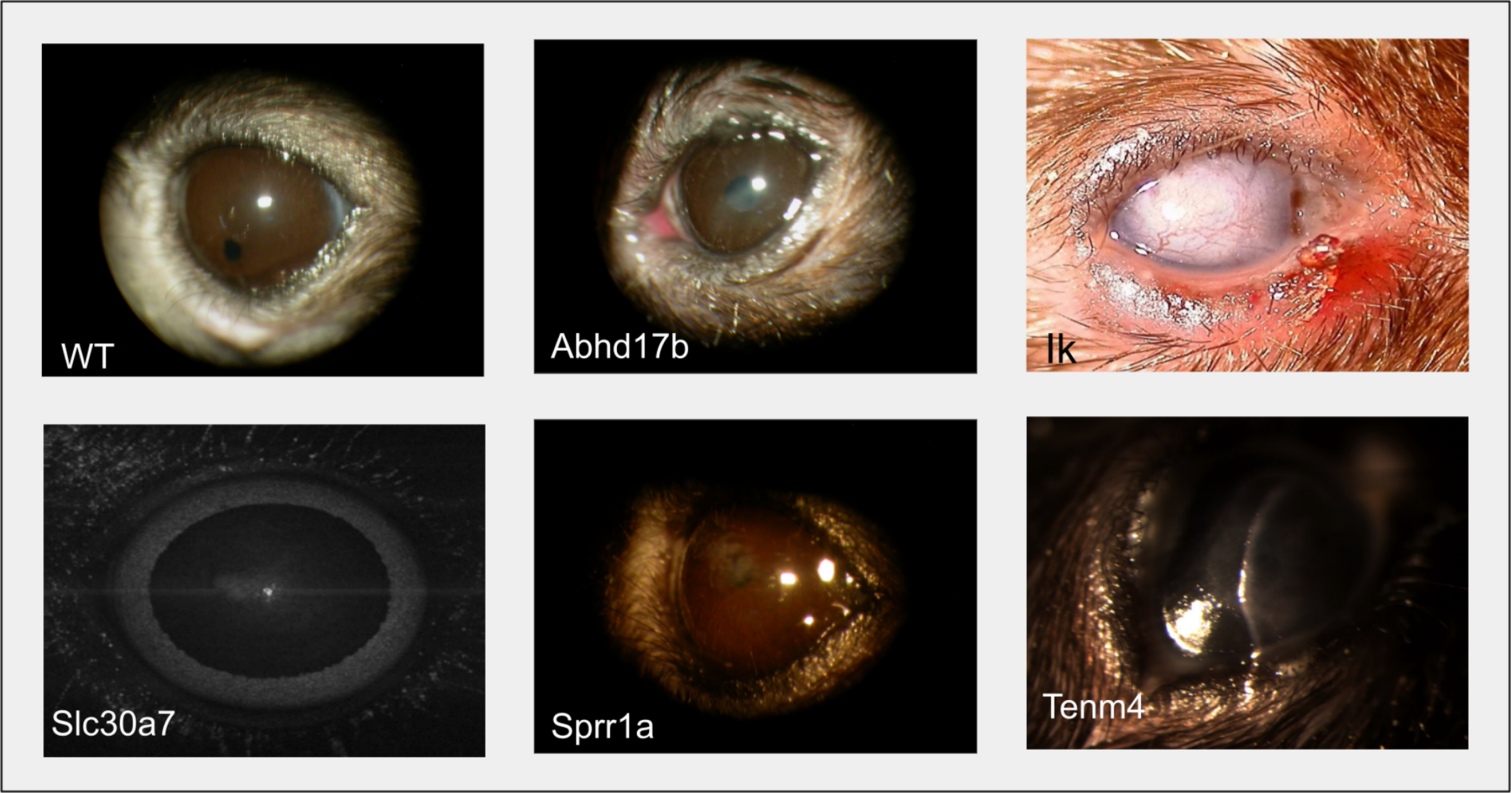
External color photography of corneas from late-stage knockout mice with documented cornea abnormalities. *Top row*: WT, *Abhd17b*^−/−^, *Ik*^+/−^. *Bottom row*: *Slc30a7*^+/−^. *Sprr1a*^−^^/^^−^, *Tenm4*^−^^/^^−^.

Of the 8901 IMPC knockout lines, 587 (7%) were evaluated in late-adult pipelines. Of those in the late-adult pipeline, 14 (2.4%) presented LACD phenotypes, which were not detected at the early-adult stage. The 14 identified LACD genes (*Abca16*, *Abhd17b*, *Fsd2*, *Galnt9*, *Gtpbp10*, *Ik*, *Krt80*, *Rgs2*, *Scamp2*, *Slc30a7*, *Sprr1a*, *Tenm4*, *Trim39*, *Vwa5a*) were characterized by ontology terms, including corneal opacity (8, 57%), increased corneal thickness (1, 7%), abnormal corneal morphology (4, 29%), and sclerocornea (1, 7%). Each of the candidate LACD genes exhibited a single corneal phenotype. Five (36%) of the 14 candidate genes had late-stage CD phenotype images available, and Figure 1 illustrates examples of these CD phenotypes compared to the wild-type cornea.

A total of 13 (93%) of the 14 candidate gene lines were homozygous knockouts, and 1 (7%) was a heterozygous knockout due to embryonic lethality in homozygotes. Moreover, six (42%) of the lines had CD phenotypes in both sexes, while the remaining eight (58%) candidate gene phenotypes achieved statistical significance in only one sex (sexual dimorphism), with six occurring in females and two in males. Nine of the gene lines (64%) displayed bilateral CD in the majority (≥50%) of mice with abnormal cornea phenotypes (*Abca16*, *Fsd2*, *Galnt9*, *Gtpbp10*, *Krt80*, *Rgs2*, *Scamp2*, *Tenm4*, *Vwa5a*), two gene lines (14%) displayed bilateral CD in the minority (<50%) of mice with abnormal corneal phenotypes (*Ik* and *Sprr1a*), two gene lines had no mice with bilateral CD (*Abhd17b* and *Trim39*), and one gene line did not have laterality data available (*Slc30a7*). For a comprehensive list of candidate LACD genes, along with their phenotypes, zygosity, sex dependence, bilaterality, and tissue expression, see Table 1.

**Table 1.** List of 14 Candidate LACD Genes With Human Orthologs, Gene Location, Full Gene Name, Associated Corneal Phenotypes, Zygosity, Gender Specificity, Life Stage, Phenotyping Center, *P* Value, Human Cornea Expression, and Previous Cornea Publication PMID

Gene (Mouse)	Ortholog (Human)	Gene Location	Gene Name	Corneal Phenotype	PLAE Human Ocular Expression	Present Bilateral	Gender Specificity	Zygosity	Life Stage	Phenotyping Center	*P* Value	Mouse PMID	Human PMID	Knockout Mouse PMID
*Abca16*	N/A	N/A	ATP-binding cassette, subfamily A (ABC1), member 16	Abnormal cornea morphology	—	Majority	Combined significant	Homozygote	Late adult	JAX	3.80E-05	—	—	—
*Abhd17b*	ABHD17B	9q21.13	Abhydrolase domain containing 17b	Abnormal cornea morphology	—	—	Combined significant	Homozygote	Late adult	JAX	5.59E-06	—	—	—
*Fsd2*	FSD2	15q25.2	Fibronectin type III and SPRY domain containing 2	Corneal opacity	—	Majority	Female	Homozygote	Late adult	UC Davis	8.89E-05	—	—	—
*Galnt9*	GALNT9	12q24.33	Polypeptide N-acetylgalactosaminyltransferase 9	Corneal opacity	—	Majority	Male	Homozygote	Late adult	MRC Harwell	3.59E-07	—	—	—
*Gtpbp10*	GTPBP10	7q21.13	GTP binding protein 10	Abnormal cornea morphology	Limbal progenitor cells (9.48%)	Majority	Combined significant	Homozygote	Late adult	JAX	2.77E-05	—	—	—
*Ik*	IK	5q31.3	IK cytokine	Abnormal cornea morphology	T/NK cells (66.67%)	Minority	Combined significant	Heterozygote	Late adult	KMPC	6.89E-05	—	—	—
*Krt80*	KRT80	12q13.13	Keratin 80	Corneal opacity	—	Majority	Male & female	Homozygote	Late adult	JAX	8.73E-11	—	Keratoconus 35821117	—
*Rgs2*	RGS2	1q31.2	Regulator of G-protein signaling 2	Corneal opacity	Corneal endothelial cells (4.79%)	Majority	Female	Homozygote	Late Adult	MRC Harwell	7.68E-05	—	—^*^	31767169
*Scamp2*	SCAMP2	15q24.1	Secretory carrier membrane protein 2	Sclerocornea	Conjunctival epithelial cells (52.19%)	Majority	Female	Homozygote	Late adult	JAX	9.45E-05	—	—	—
*Slc30a7*	SLC30A7	1p21.2	Solute carrier family 30 member 7	Increased corneal thickness	T/NK cells (33.33%)	N/A	Male	Homozygote	Late adult	BCM	2.23E-05	—	—	29555680
*Sprr1a*	SPRR1A/SPRR1B	1q21.3	Small proline-rich protein 1A	Corneal opacity	—	Minority	Female	Homozygote	Late adult	JAX	7.97E-06	22673847	Keratoconus 36240204	—
*Tenm4*	TENM4	11q14.1	Teneurin transmembrane protein 4	corneal opacity	Corneal progenitor cells (32.41%)	Majority	Female	Homozygote	Late adult	TCP	7.18E-07	—	—	37092850
*Trim39*	TRIM39	6p22.1	Tripartite motif-containing 39	Corneal opacity	—	—	Combined significant	Homozygote	Late adult	MRC Harwell	3.27E-05	—	—	—
*Vwa5a*	VWA5A	11q24.2	von Willebrand factor A domain containing 5A	Corneal opacity	Conjunctival Epithelium (26.04%)	Majority	Female	Homozygote	Late adult	JAX	5.16E-05	—	—	—

* https://iovs.arvojournals.org/article.aspx?articleid=2418919.

Two genes (*Fsd2* and *Scamp2*) found to have LACD in late adulthood exhibited other abnormal ocular phenotypes in early adulthood. Specifically, *Fsd2* showed an early-adult “abnormal eye morphology” phenotype, and *Scamp2* presented early-adult “cataracts.”

A thorough literature search revealed two of the candidate genes (*Krt80* and *Sprr1A*) had been previously associated with an existing CD, specifically keratoconus (KCN).^26^^,^^27^ The remaining 12 candidate LACD genes were novel, given that they had no prior literature associating them with CDs (*Abca16*, *Abhd17b*, *Fsd2*, *Galnt9*, *Gtpbp10*, *Ik*, *Rgs2*, *Scamp2*, *Slc30a7*, *Tenm4*, *Trim39*, *Vwa5a*).

Seven of the 14 LACD genes (*Gtpbp10*, *Ik*, *Rgs2*, *Scamp2*, *Slc30a7*, *Tenm4*, *Vwa5a*) (50%) were found expressed in single-cell transcriptomic data sets from the human cornea using PLAE. For comparison, 34 of the 48 (71%) established human CD genes studied in a recent publication were found expressed using PLAE19. These genes were expressed in multiple corneal cell types; GTPBP10 is expressed most in limbal progenitor cells along with 12 additional cell types, Ik is expressed most in T/natural killer cells along with 18 additional cell types, RGS2 is expressed most in corneal endothelial cells along with 9 additional cell types, SCAMP2 is expressed most in conjunctival epithelium along with 17 additional cell types, SLC30A7 is expressed most in T/natural killer cells along with 17 additional cell types, TENM4 is expressed most in corneal progenitor cells along with 15 additional cell types, and VWA5A is expressed most in conjunctival epithelial cells along with 12 additional cell types. For a comprehensive list of candidate LACD gene expression, see Table 2.

**Table 2.** List of Seven LACD Genes Expressed in Human Corneal Tissue on PLAE With Cell Type, Cell Expression Count, Cell Count, Percent Expression, and Overall Expression

Gene	Cell Type	cell_exp_ct	Count	%	Expression
LACD genes expressed in human cornea on PLAE					
*GTPBP10*	Blood vessel	53	1013	5.23	0.049054606
	Conjunctival epithelial	68	914	7.44	0.064204752
	Corneal endothelial	56	906	6.18	0.05563471
	Corneal epithelial	70	1443	4.85	0.042205409
	Corneal nerve	18	227	7.93	0.078659697
	Corneal progenitor	146	2243	6.51	0.05554956
	Fibroblast	719	16,716	4.3	0.037233542
	Keratocyte	1233	31,850	3.87	0.03640526
	Limbal	61	2040	2.99	0.02548333
	Limbal progenitor	11	116	9.48	0.062033789
	Melanocyte	14	322	4.35	0.040859916
	Mesoderm	46	718	6.41	0.060610513
	Proliferating cornea	216	4184	5.16	0.047236267
*IK*	Blood vessel	457	1013	45.11	0.60722162
	Ciliary margin	96	366	26.23	0.303617618
	Conjunctival epithelial	572	914	62.58	0.844592509
	Corneal basement membrane	50	316	15.82	0.113733385
	Corneal endothelial	500	906	55.19	0.718289442
	Corneal epithelial	545	1443	37.77	0.420991108
	Corneal nerve	110	227	48.46	0.570850791
	Corneal progenitor	1256	2243	56	0.677466004
	Fibroblast	7827	16,716	46.82	0.509755583
	Keratocyte	14,419	31,850	45.27	0.523381554
	Limbal	856	2040	41.96	0.456651773
	Limbal Progenitor	56	116	48.28	0.477717703
	Melanocyte	186	322	57.76	0.696180743
	Mesoderm	403	718	56.13	0.7084185
	Monocyte	2	9	22.22	0.217136975
	Neural crest	750	4030	18.61	0.167899486
	Proliferating cornea	2263	4184	54.09	0.661015996
	Red blood cell	83	446	18.61	0.09014182
	T/NK cell	2	3	66.67	1.193903713
*RGS2*	Corneal endothelial	134	906	14.79	0.16677905
	Corneal nerve	8	227	3.52	0.031892219
	Corneal progenitor	115	2243	5.13	0.048288945
	Fibroblast	540	16,716	3.23	0.029757948
	Keratocyte	760	31,850	2.39	0.021673073
	Limbal	50	2040	2.45	0.018007651
	Melanocyte	8	322	2.48	0.020341751
	Mesoderm	77	718	10.72	0.109691282
	Monocyte	1	9	11.11	0.110818462
	Proliferating cornea	159	4184	3.8	0.035409881
*SCAMP2*	Blood vessel	332	1013	32.77	0.407705583
	Ciliary margin	10	366	2.73	0.025379864
	Conjunctival epithelial	477	914	52.19	0.729825422
	Corneal basement membrane	39	316	12.34	0.095550319
	Corneal endothelial	199	906	21.96	0.224416227
	Corneal epithelial	253	1443	17.53	0.174961006
	Corneal nerve	50	227	22.03	0.235026946
	Corneal progenitor	558	2243	24.88	0.246266716
	Fibroblast	4480	16,716	26.8	0.269089102
	Keratocyte	8527	31,850	26.77	0.284728093
	Limbal	542	2040	26.57	0.275048788
	Limbal progenitor	33	116	28.45	0.246732869
	Melanocyte	94	322	29.19	0.281353239
	Mesoderm	181	718	25.21	0.243828375
	Monocyte	1	9	11.11	0.174674709
	Neural crest	416	4030	10.32	0.09467133
	Proliferating cornea	1196	4184	28.59	0.292412064
	Red blood cell	51	446	11.43	0.052587223
*SLC30A7*	Blood vessel	269	1013	26.55	0.308102919
	Ciliary margin	19	366	5.19	0.057439352
	Conjunctival epithelial	241	914	26.37	0.262958797
	Corneal basement membrane	32	316	10.13	0.085788164
	Corneal endothelial	204	906	22.52	0.227424494
	Corneal epithelial	180	1443	12.47	0.111259617
	Corneal nerve	47	227	20.7	0.201737179
	Corneal progenitor	512	2243	22.83	0.228073599
	Fibroblast	3998	16,716	23.92	0.245216334
	Keratocyte	8020	31,850	25.18	0.275891635
	Limbal	405	2040	19.85	0.187106687
	Limbal progenitor	18	116	15.52	0.10392457
	Melanocyte	72	322	22.36	0.241311863
	Mesoderm	197	718	27.44	0.306550095
	Neural crest	427	4030	10.6	0.097545113
	Proliferating cornea	1226	4184	29.3	0.314065322
	Red blood cell	28	446	6.28	0.025026106
	T/NK cell	1	3	33.33	0.332920908
*TENM4*	Blood vessel	48	1013	4.74	0.027767808
	Conjunctival epithelial	70	914	7.66	0.056175485
	Corneal basement membrane	56	316	17.72	0.133070722
	Corneal endothelial	185	906	20.42	0.247310964
	Corneal epithelial	137	1443	9.49	0.060927395
	Corneal nerve	28	227	12.33	0.078367014
	Corneal progenitor	727	2243	32.41	0.345418154
	Fibroblast	2168	16,716	12.97	0.11951044
	Keratocyte	6715	31,850	21.08	0.217220265
	Limbal	201	2040	9.85	0.088300576
	Limbal progenitor	21	116	18.1	0.149686569
	Melanocyte	21	322	6.52	0.049998262
	Mesoderm	107	718	14.9	0.142818617
	Neural crest	654	4030	16.23	0.15439657
	Proliferating cornea	857	4184	20.48	0.20501373
	Red blood cell	26	446	5.83	0.016273841
*VWA5A*	Blood vessel	31	1013	3.06	0.019780834
	Conjunctival epithelial	238	914	26.04	0.283712544
	Corneal endothelial	74	906	8.17	0.080988958
	Corneal epithelial	60	1443	4.16	0.035334629
	Corneal nerve	9	227	3.96	0.034558655
	Corneal progenitor	180	2243	8.02	0.069566317
	Fibroblast	516	16,716	3.09	0.026088103
	Keratocyte	1016	31,850	3.19	0.028529178
	Limbal	177	2040	8.68	0.077407876
	Limbal progenitor	6	116	5.17	0.049003377
	Mesoderm	51	718	7.1	0.066045241
	Proliferating cornea	164	4184	3.92	0.033099922
	Red blood cell	10	446	2.24	0.014846217

STRING protein analysis within Cytoscape, excluding *Abca16* (since it has no human ortholog) and *MIR184* (microRNA) from the candidate and established CD gene sets, respectively, found no protein interactions within the candidate gene set (data not shown). When including 10 additional interactor proteins in the candidate gene analysis, four functional clusters emerged in the highest confidence interval (>0.9) containing four candidate genes (*Ik*, *Rgs2*, *Sprr1a*, *Tenm4*), as seen in Supplemental Figure S1. Analysis at the high confidence interval included the gene *Trim39* into one of the four clusters, while analysis at the moderate confidence interval resulted in the inclusion of three more candidate genes (*Fsd2*, *Krt80*, *Slc30a7*) into five total clusters.

**Figure 2.**
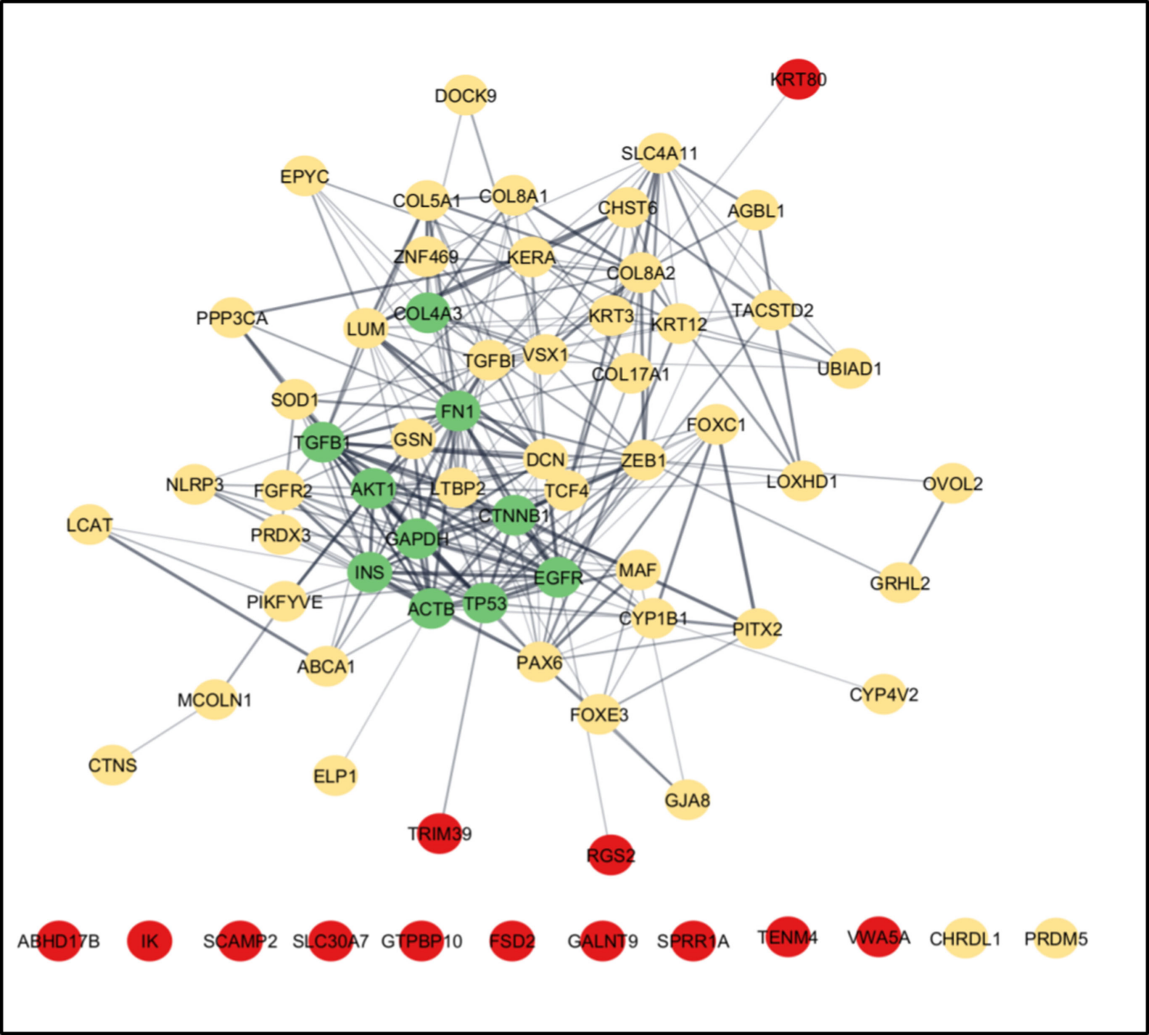
STRING protein–protein analysis between human ortholog proteins of 13 candidate LACD genes (*red*), 47 established human CD proteins (*gold*), and 10 additional interactor proteins determined by STRING (*green*). Candidate LACD gene *Abca16* and established human CD gene *MIR-184* were omitted from this analysis as they are not available in STRING. Analysis run with modified settings (Organism: Homo Sapiens; Network Type = full STRING network; Confidence cutoff 0.40; Additional interactors 10). Darker edges indicate stronger protein–protein interaction.

Protein interactions between candidate LACD genes and established human CD genes revealed no protein clusters involving candidate genes at the highest or high confidence level. One protein cluster containing a candidate gene (*Krt80*) emerged in the moderate confidence interval (Supplemental Fig. S2). When including 10 additional interactor proteins in the candidate and established CD gene analysis, one protein cluster containing three candidate genes (*Krt80*, *Rgs2*, *Trim39*) emerged in the moderate confidence interval (Fig. 2).

Protein interactions between candidate LACD genes and genes that resulted in early-adult CD via the IMPC^19^ revealed one cluster containing one candidate gene (*Ik*) in the highest and high confidence interval. Three clusters emerged containing three additional candidate genes (*Rgs2*, *Slc30a7*, *Sprr1a*) at the moderate confidence interval, as seen in Supplemental Figure S3. A detailed list of genes with corresponding protein–protein confidence levels can be found in Table 3.

Protein interactions of each of the seven LACD genes expressed in the human cornea returned four genes with protein networks (*Gtpbp10*, *Ik*, *Rgs2*, *Tenm4*), while three genes (*Scamp2*, *Slc30a7*, *Vwa5a*) did not have any protein networks at the >0.90 confidence level. *Gtpbp10* had 10 protein interactions, including mitochondrial ribosomal assembly proteins; *Ik* had 10 protein interactions, including pre-mRNA splicing proteins; *Rgs2* had 5 protein interactions, including G-protein signaling proteins; and *Tenm4* had 3 protein interactions, including cell–cell adhesion proteins, as seen in Supplemental Figure S4.

**Figure 3.**
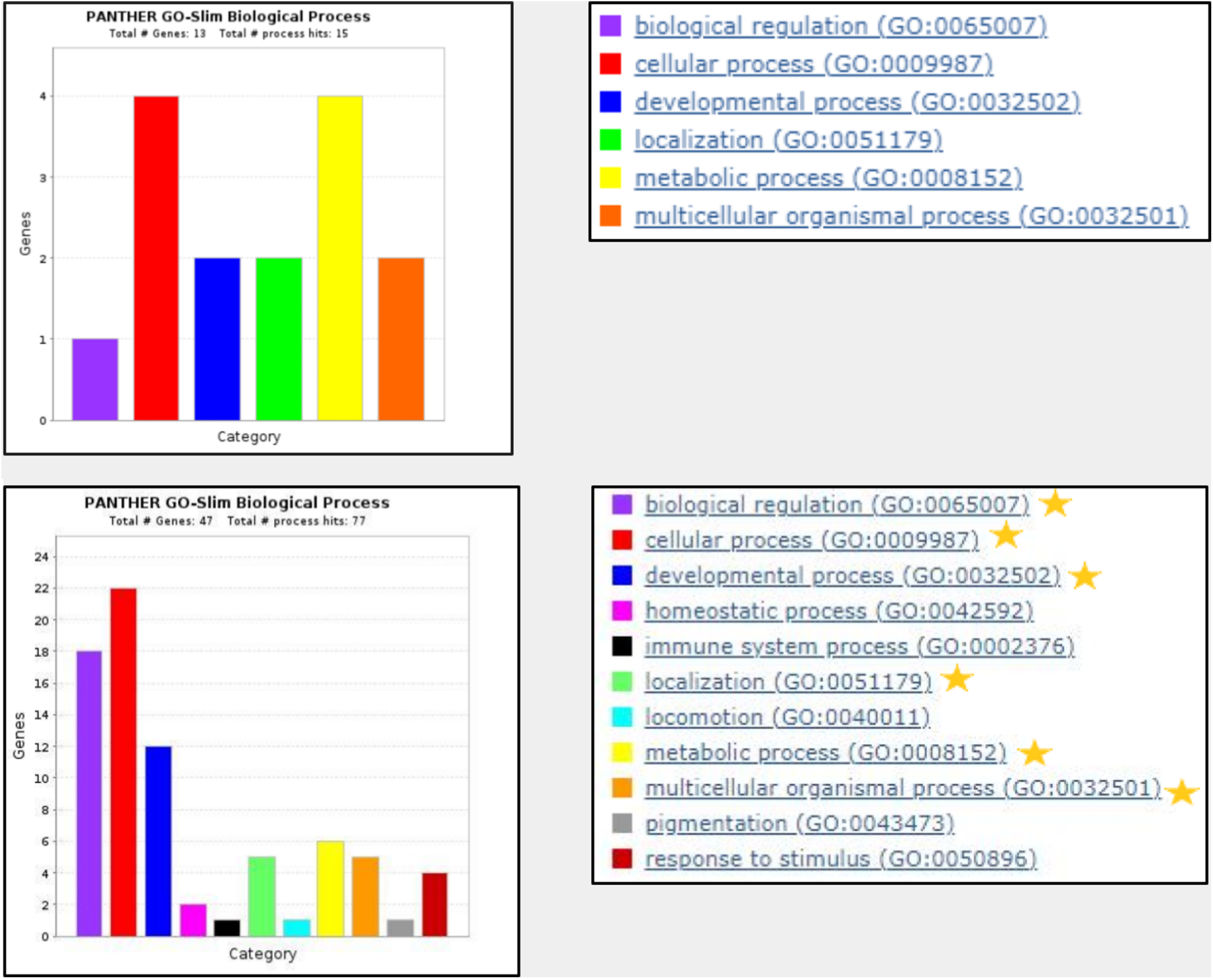
Biological process categories of 13 candidate LACD genes (*top*) and 47 established human CD genes (*bottom*) using PANTHER analysis. Candidate LACD gene *Abca16* and established human CD gene *MIR-184* were not included in the analysis as they were not available on PANTHER. *Gold star* depicts biological processes found in both candidate LACD genes and established CD genes.

In PANTHER biological process analysis, 13 of the 14 candidate LACD genes (excluding *Abca16*) were mapped to 6 biological process categories, and 47 of the 48 established human CD genes (excluding *MIR-184*) were mapped to 11 biological process categories, as seen in Figure 3. All six biological processes categories were shared between the two gene sets (biological regulation, cellular process, developmental process, localization, metabolic process, and multicellular organismal process), while the established human CD genes had five unique biological process categories (homeostatic process, immune system process, locomotion, pigmentation, and response to stimulus). A detailed list associating candidate and established genes with specific biological processes can be found in Table 4.

**Table 3.** Confidence Ranking of STRING Protein-Protein Interactions Among Candidate LACD Genes, and Between Candidate LACD Genes and Established Human CD Genes, Early Adult-Stage CD Genes, and 10 Additional STRING Interactor Proteins.

	Candidate Gene Protein–Protein Interaction Confidence Score				
Gene Set	Highest (1.00–0.90)	High (0.90–0.70)	Medium (0.70–0.40)	Low (0.40–0.15)	No Interactions (<0.15)
Late stage	—	—	—	SPRR1A	ABCA16
				KRT80	ABHD17B
					FSD2
					GALNT9
					GTPBP10
					IK
					RGS2
					SCAMP2
					SLC30A7
					TENM4
					TRIM39
					VWA5A
Late stage + 10 interactors	IK	TRIM39	KRT80	ABHD17b	GALNT9
	TENM4		FSD2	GTPBP10	SCAMP2
	SPRR1A		SLC30A7		
	RGS2				
Late stage + established human CD genes	—	—	KRT80	FSD2	ABCA16
				IK	ABHD17B
				SPRR1A	GALNT9
				VWA5A	RGS2
				TENM4	SCAMP2
				SLC30A7	TRIM39
				GTPBP10	
Late stage + established human CD genes (+10 interactors)	—	—	KRT80	IK	ABCA16
			TRIM39	TENM4	ABHD17B
				RGS2	GALNT9
				FSD2	
				SLC30A7	
				SPRR1A	
				VWA5A	
				SCAMP2	
				GTPBP10	
Late stage + early stage	IK	—	SPRR1A	ABHD17B	ABCA16
	TENM4		RGS2	FSD2	
	SPRR1A		SLC30A7	GALNT9	
	RGS2			GTPBP10	
				TENM4	
				TRIM39	
				VWA5A	
				KRT80	
				SCAMP2	

**Figure 4.**
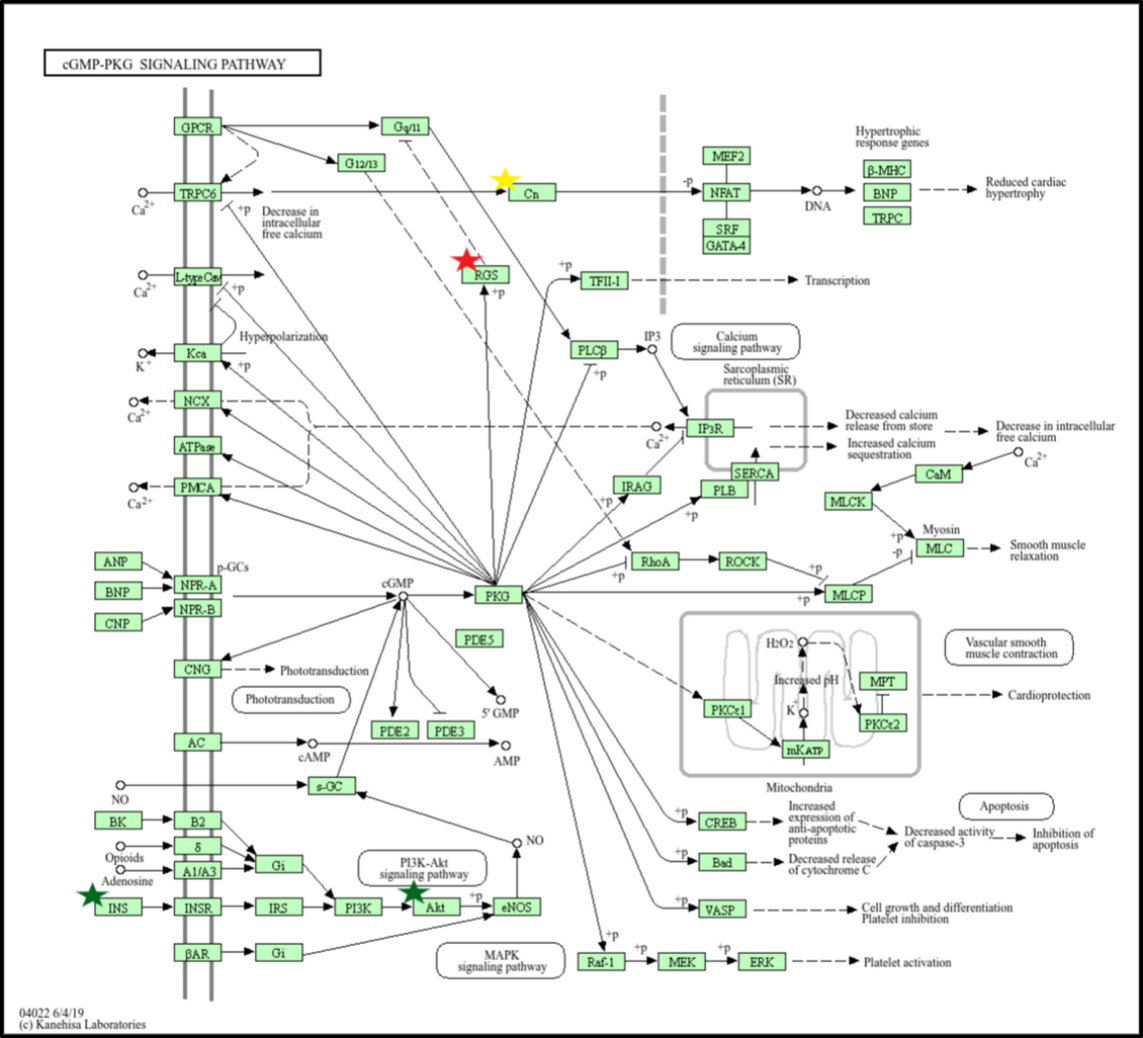
cGMP-PKG signaling pathway highlighting candidate LACD gene *Rgs2* (*red star*), established CD genes *CNA1* (*gold star*), and two additional STRING interactor genes *INS* and *AKT1* (*green star*).

KEGG pathway mapping of the 14 candidate LACD genes, the established human CD genes, and the 10 additional interactor genes was combined, resulting in three pathways containing candidate LACD genes: cGMP-PKG signaling, oxytocin signaling, and mucin-type O-glycan synthesis. The cGMP signaling pathway (Fig. 4) contains one candidate LACD gene (*Rgs2*), one established gene (*Cna1*), and two interactor genes (*Ins* and *Akt1*). The oxytocin signaling pathway (Supplemental Fig. S4) includes LACD gene *Rgs2* and two interactors (*Egfr* and *Actb*). The mucin-type O-glycan biosynthesis pathway (Supplemental Fig. S5) includes LACD gene *Galnt9*. Five additional pathways did not contain candidate LACD genes but contained established CD genes or additional interactor genes found to have protein–protein interactions with the candidate LACD gene set, MAPK signaling, WNT signaling, signaling pathways regulating pluripotency of stem cells, regulation of actin cytoskeleton, and cellular senescence. These can be viewed in Supplemental Figures S6 to S10. A full list of KEGG pathways for candidate and established CD genes can be found in Table 5.

**Table 4.** List of PANTHER Biological Processes Associated With Candidate LACD Genes (Left) and Established Human CD Genes (Right)

Gene	PANTHER (Biological Process)	Gene (Established Human CD Genes)	PANTHER (Biological Process)
*Abca16*	—	*Abca1*	Phospholipid transport
*Abhd17b*	Regulation of postsynapse organization	*Agbl1*	Peptidyl-amino acid modification
	Protein modification process		
	Protein catabolic process		
	Lipoprotein metabolic process		
*Fsd2*	—	*Chrdl1*	Cell differentiation
			Negative regulation of BMP signaling pathway
*Galnt9*	Protein O-linked glycosylation	*Chst6*	Sulfur compound metabolic process
			Amino sugar metabolic process
*Gtpbp10*	—	*Cna1*	Inositol phosphate-mediated signaling
			Calcium-mediated signaling
*Ik*	mRNA splicing, via spliceosome	*Col17a1*	Extracellular matrix organization
*Krt80*	Intermediate filament organization*	*Col5a1*	Extracellular matrix organization
	Skin development*		
	Multicellular organismal process*		
	Epidermal cell differentiation*		
*Rgs2*	—	*Col8a1*	Extracellular matrix organization
*Scamp2*	Protein transport	*Col8a2*	Extracellular matrix organization
*Slc30a7*	Metal ion transport	*Ctns*	L-amino acid transport
			Neutral amino acid transport
*Sprr1a*	—	*Cyp1b1*	—
*Tenm4*	Heterophilic cell–cell adhesion via plasma membrane cell adhesion molecules	*Cyp4v2*	—
	Neuron development		
*Trim39*	Protein ubiquitination	*Dcn*	—
*Vwa5a*	—	*Dock9*	Positive regulation of GTPase activity
		*Epyc*	Cartilage development
			Bone development
		*Fgfr2*	Multicellular organism development
			Transmembrane receptor protein tyrosine kinase signaling pathway
			Positive regulation of kinase activity
		*Foxc1*	Anatomical structure morphogenesis
			Regulation of transcription by RNA polymerase II
			Cell differentiation
		*Foxe3*	Anatomical structure morphogenesis
			Regulation of transcription by RNA polymerase II Cell differentiation
		*Gja8*	Cell–cell signaling
		*Grhl2*	Chordate embryonic development
			Brain development
			Epithelium development
			Regulation of transcription by RNA polymerase II
			Tube development
		*Gsn*	Barbed-end actin filament capping
			Central nervous system development
			Cell projection assembly
			Actin polymerization or depolymerization
		*Ikbkap*	tRNA wobble uridine modification
		*Kera*	—
		*Krt12*	Intermediate filament organization*
			Epithelial cell differentiation
		*Krt3*	Multicellular organismal process*
			Intermediate filament organization*
			Epidermal cell differentiation*
			Skin development*
		*Lcat*	Lipid metabolic process
		*Loxhd1*	—
		*Ltbp2*	Supramolecular fiber organization
		*Lum*	—
		*Maf*	Regulation of transcription by RNA polymerase II
		*Mcoln1*	—
		*miR-184*	—
		*Nlrp3*	Regulation of inflammatory response
		*Ovol2*	Regulation of transcription by RNA polymerase II
			Epidermal cell differentiation
		*Pax6*	Anatomical structure development
			Regulation of transcription by RNA polymerase II
		*Pikfyve*	Regulation of cellular metabolic process
			Melanosome organization
			Phagolysosome assembly
			Neutrophil chemotaxis
			Vesicle fusion
			Regulation of biosynthetic process
		*Pitx2*	Anatomical structure morphogenesis
			Regulation of transcription by RNA polymerase II
		*Prdm5*	Negative regulation of DNA-templated transcription
		*Prdx3*	Cellular homeostasis
			Response to oxidative stress
			Cellular response to stress
			Organic substance catabolic process
			Cellular catabolic process
		*Slc4a11*	Transmembrane transport
			Monoatomic ion homeostasis
		*Sod1*	—
		*Tacstd2*	—
		*Tcf4*	Regulation of transcription by RNA polymerase II
		*Tgfbi*	Cell adhesion
			Extracellular matrix organization
		*Ubiadi*	Ubiquinone biosynthetic process
		*Vsx1*	Regulation of DNA-templated transcription
		*Zeb1*	—
		*Znf469*	—

Processes shared between gene sets are denoted by *.

**Table 5.** List of KEGG Pathways for Candidate LACD Genes and Established CD Genes

Gene	KEGG Pathway	Pathway ID
**Candidate CD Genes**		
*Abca16*	—	—
*Abhd17b*	—	—
*Fsd2*	—	—
*Galnt9*	Mucin type O-glycan biosynthesis	hsa00512
	Metabolic pathways*	hsa01100
	Other types of O-glycan biosynthesis	hsa00514
*Gtpbp10*	—	—
*Ik*	—	—
*Krt80*	—	—
*Rgs2*	cGMP-PKG signaling pathway*	hsa04022
	Oxytocin signaling pathway*	hsa04921
	Olfactory transduction	hsa04740
*Scamp2*	—	—
*Slc30a7*	—	—
*Sprr1a*	—	—
*Tenm4*	—	—
*Trim39*	—	—
*Vwa5a*	—	—
**Established Human CD Genes**		
*Abca1*	ABC transporters	hsa02010
	Fat digestion and absorption	hsa04975
	Cholesterol metabolism	hsa04979
	Lipid and atherosclerosis	hsa05417
	Efferocytosis	hsa04148
*Agbl1*	—	—
*Chrdl1*	—	—
*Chst6*	Glycosaminoglycan biosynthesis—keratan sulfate	hsa00533
*Cna1*	MAPK signaling pathway	hsa04010
	Calcium signaling pathway	hsa04020
	cGMP-PKG signaling pathway*	hsa04022
	Oocyte meiosis	hsa04114
	Cellular senescence	hsa04218
	Wnt signaling pathway	hsa04310
	Axon guidance	hsa04360
	VEGF signaling pathway	hsa04370
	Osteoclast differentiation	hsa04380
	C-type lectin receptor signaling pathway	hsa04625
	Natural killer cell–mediated cytotoxicity	hsa04650
	Th1 and Th2 cell differentiation	hsa04658
	Th17 cell differentiation	hsa04659
	T-cell receptor signaling pathway	hsa04660
	B-cell receptor signaling pathway	hsa04662
	Long-term potentiation	hsa04720
	Glutamatergic synapse	hsa04724
	Dopaminergic synapse	hsa04728
	Oxytocin signaling pathway*	hsa04921
	Glucagon signaling pathway	hsa04922
	Renin secretion	hsa04924
	Alzheimer disease	hsa05010
	Amyotrophic lateral sclerosis	hsa05014
	Prion disease	hsa05020
	Pathways of neurodegeneration—multiple diseases	hsa05022
	Amphetamine addiction	hsa05031
	Tuberculosis	hsa05152
	Human cytomegalovirus infection	hsa05163
	Human T-cell leukemia virus 1 infection	hsa05166
	Kaposi sarcoma–associated herpesvirus infection	hsa05167
	Human immunodeficiency virus 1 infection	hsa05170
	PD-L1 expression and PD-1 checkpoint pathway in cancer	hsa05235
	Lipid and atherosclerosis	hsa05417
*Col17a1*	Protein digestion and absorption	hsa04974
*Col5a1*	Protein digestion and absorption	hsa04974
*Col8A1*	Protein digestion and absorption	hsa04974
*Col8a2*	Protein digestion and absorption	hsa04974
*Ctns*	Lysosome	hsa04142
*Cyp1b1*	Steroid hormone biosynthesis	hsa00140
	Tryptophan metabolism	hsa00380
	Metabolism of xenobiotics by cytochrome P450	hsa00980
	Ovarian steroidogenesis	hsa04913
	Chemical carcinogenesis—DNA adducts	hsa05204
	MicroRNAs in cancer	hsa5206
	Chemical carcinogenesis—receptor activation	hsa05207
	Chemical carcinogenesis—reactive oxygen species	hsa05208
*CYP4V2*	—	—
*Dcn*	TGF-beta signaling pathway	hsa04350
	Proteoglycans in cancer	hsa05205
	Cytoskeleton in muscle cells	hsa04820
*Dock9*	—	—
*Epyc*	—	—
*Fgfr2*	EGFR tyrosine kinase inhibitor resistance	hsa01521
	MAPK signaling pathway	hsa04010
	Ras signaling pathway	hsa04014
	Rap1 signaling pathway	hsa04015
	Calcium signaling pathway	hsa04020
	Endocytosis	hsa04144
	PI3K-Akt signaling pathway	hsa04151
	Signaling pathways regulating pluripotency of stem cells	hsa04550
	Regulation of actin cytoskeleton	hsa04810
	Pathways in cancer	hsa05200
	Prostate cancer	hsa05215
	Gastric cancer	hsa05226
	Central carbon metabolism in cancer	hsa05230
*Foxc1*	—	—
*Foxe3*	—	—
*Gja8*	—	—
*Grhl2*	—	—
*Gsn*	Regulation of actin cytoskeleton	hsa04810
	Fc gamma R-mediated phagocytosis	hsa04666
	Viral carcinogenesis	hsa05203
*Ikbkap*	—	—
*Kera*	—	—
*Krt12*	Estrogen signaling pathway	hsa04915
	*Staphylococcus aureus* infection	hsa05150
*Krt3*	—	—
*Lcat*	Cholesterol metabolism	hsa04979
	Glycerophospholipid metabolism	hsa00564
*Loxhd1*	—	—
*Ltbp2*	—	—
*Lum*	Proteoglycans in cancer	hsa05205
*Maf*	Th1 and Th2 cell differentiation	hsa04658
	Transcriptional misregulation in cancer	hsa05202
	Inflammatory bowel disease	
*Mcoln1*	Lysosome	hsa04142
	Calcium signaling pathway	hsa04020
*miR-184*	—	—
*Nlrp3*	Necroptosis	hsa04217
	NOD-like receptor signaling pathway	hsa04621
	Cytosolic DNA-sensing pathway	hsa04623
	C-type lectin receptor signaling pathway	hsa04625
	Salmonella infection	hsa05132
	Pertussis	hsa05133
	Yersinia infection	hsa05135
	Influenza A	hsa05164
	Coronavirus disease—COVID-19	hsa05171
	Lipid and atherosclerosis	hsa05417
	Pathogenic *Escherichia coli* infection	hsa05130
	Shigellosis	hsa05131
*Ovol2*	—	—
*Pax6*	Signaling pathways regulating pluripotency of stem cells	hsa04550
	Maturity-onset diabetes of the young	hsa04950
*Pikfyve*	Inositol phosphate metabolism	hsa00562
	Metabolic pathways*	hsa01100
	Phosphatidylinositol signaling system	hsa04070
	Phagosome	hsa04145
	Regulation of actin cytoskeleton	hsa04810
*Pitx2*	TGF-beta signaling pathway	hsa04350
*Prdm5*	—	—
*Prdx3*	—	—
*Slc4a11*	—	—
*Sod1*	Peroxisome	hsa04146
	Longevity regulating pathway—multiple species	hsa04213
	Parkinson disease	hsa05012
	Amyotrophic lateral sclerosis	hsa05014
	Huntington disease	hsa05016
	Prion disease	hsa05020
	Pathways of neurodegeneration—multiple diseases	hsa05022
	Chemical carcinogenesis—reactive oxygen species	hsa05208
*Tacstd2*	—	—
*Tcf4*	—	—
*Tgfbi*	—	—
*Ubiadi*	—	—
*Vsx1*	—	—
*Zeb1*	Transcriptional misregulation in cancer	hsa05202
	MicroRNAs in cancer	hsa05206
	Prostate cancer	hsa05215
*Znf469*	—	—

Processes shared between gene sets are denoted by *.

## Discussion

In this study, we identified 14 mammalian genes required for corneal clarity in the late-adult phase (***Abca16***, ***Abhd17b***, ***Fsd2***, ***Galnt9***, ***Gtpbp10***, ***Ik***, *Krt80*, ***Rgs2***, ***Scamp2***, ***Slc30a7***, *Sprr1a*, ***Tenm4***, ***Trim39***, ***Vwa5a***). Most of these genes, 12 of 14 (bold above), have no reported functional roles in corneal biology. While half of the 14 candidate genes are expressed in human cornea, most of them do not have obvious bioinformatic relationships with established CD genes and may have biological functions that are not well understood.

The seven candidate genes expressed in human cornea tissue presented in multiple cornea cell types with multiple cell types shared among the genes. Given this overlap in expression, a direct relationship of cell type expressed to observed phenotype is not easily derived. Furthermore, the protein networks generated from the expressed genes do not present a clear relationship of protein network to observed phenotype.

Examining the functions of these genes outside the cornea provides insight into their potential roles within it. Gtpbp10, a GTP-binding protein, aids in mitochondrial ribosomal RNA folding.^28^ This function is supported by its high-confidence interactions with mitochondrial ribosomal assembly proteins in STRING, suggesting a similar role in the aged cornea, particularly in limbal progenitor cells, where its expression is highest. Ik, a cytokine, inhibits interferon-gamma–induced major histocompatibility complex class II expression and is a spliceosome component in noncorneal tissues.^29^^,^^30^ STRING analysis revealed interactions only with other spliceosome proteins. However, its highest expression in corneal T/natural killer cells suggests its immune-modulating functions may contribute to the abnormal corneal morphology observed. Scamp2, a secretory carrier membrane protein, facilitates post-Golgi recycling and regulates cell surface T-type calcium channels in noncorneal tissues.^31^^,^^32^ STRING did not reveal high-confidence protein interactions, and its highest expression in conjunctival epithelial cells does not indicate a clear corneal function in this analysis. Slc30a7, a zinc transporter, enables cellular zinc efflux and has demonstrated antioxidant effects in high-glucose apoptosis outside the cornea.^33^^,^^34^ It had no high-confidence interactions in STRING and was most highly expressed in T/natural killer cells. The observed LACD phenotype may stem from disruptions in zinc homeostasis, antioxidant effects, or an unknown mechanism. Tenm4, a teneurin transmembrane protein, promotes focal adhesion kinase activation and interacts with adhesion G protein-coupled receptors.^35^ It is most highly expressed in corneal progenitor cells, suggesting that impaired cell–cell adhesion in these cells could disrupt corneal architecture, potentially leading to corneal opacity. Vwa5a, a von Willebrand factor A domain-containing protein, may function as a tumor suppressor outside the cornea.^36^ Protein analysis provided little insight, but its highest expression in conjunctival epithelium raises the possibility that the observed corneal opacity may result from conjunctival overgrowth.

*Rgs2*, a regulator of G-protein signaling, emerges as a noteworthy candidate LACD gene with multiple pathways and interactions that could explain its corneal opacity phenotype in knockout mouse lines. The cGMP-PKG pathway has been previously linked to regulation of collagen synthesis in multiple organs, including the eye, but has not yet been implicated in CD.^7^ Due to the importance of collagen in maintaining the integrity and physiological properties of the corneal stroma, it follows that a disruption of the collagen architecture in an aging cornea could compromise its translucent properties, resulting in corneal opacity. Given the direct involvement of *Rgs2* in this cGMP-PKG pathway, the knockout of this gene may compromise the pathway enough to result in a CD. Furthermore, *Rgs2* is directly involved in oxytocin signaling, a pathway implicated in dry eye disease (DED).^9^ It is known that DED can result in corneal epithelial opacity; therefore, *Rgs2* knockout and subsequent phenotypic corneal opacity could also be explained through this relationship. *Rgs2* also had indirect involvement in other signaling pathways such as MAPK, WNT, and regulation of pluripotency of stem cells signaling; however, these interactions were of lower confidence. It is unclear if the *Rgs2* knockout and emergent corneal opacity phenotype is a result of disruption to one or more of the previously mentioned pathways.

Several other candidate LACD genes were similarly found to result in corneal opacities. One such gene is *Galnt9*, polypeptide N-acetylgalactosaminyltransferase 9, found in the mucin-type O-glycan biosynthesis pathway. It is well established that mucin plays a vital role in protecting the cornea, and its disruption can result in epithelial damage.^8^ Therefore, it follows that disruption to the synthesis of the mucin layer would leave the cornea susceptible to damage, resulting in corneal opacity. *Trim39*, tripartite motif-containing 39, is another candidate LACD gene resulting in a corneal opacity phenotype. Disruption of the p53 pathway has been shown to affect the differentiation and mucin expression of corneal epithelial cells.^37^ Therefore, *Trim39*’s moderate interaction with notable interactor gene Tp53 within the cellular senescence pathway could represent a link to corneal mucin production. Compromise in mucin expression and cell turnover, especially in the rapidly replicating corneal epithelial cells, could allow damaged cells to accumulate and disrupt the translucent nature of this tissue layer, resulting in corneal opacities. Both *Galnt9* and *Trim39*’s phenotypic effects on corneal opacity highlight the importance that mucin expression and proper epithelial function have on the overall integrity and function of the cornea.

The analysis of late-stage CD genes is incomplete due to the consortium's ability to include only ∼7% of IMPC knockout lines for aging phenotypes. Furthermore, our analysis does not span the whole mouse genome, as it is based on data from the IMPC, which includes 8901 protein-coding genes. Only a fraction of these genes underwent late-adult phenotyping. Of the genes labeled with cornea phenotypes, some false positives existed due to incomplete data entry at the IMPC data portal. Since the IMPC data set is dynamic with new data releases every 3 to 4 months, the nature of this research demands independent verification. This study is based on high-throughput ocular examination by expert graders who are masked to the genotype of mice. However, due to limitations in scope and funding, their findings were not always assessed by histopathology. *Ik* and *Sprr1a* displayed bilateral corneal phenotypes in less than 50% of mice. *Abhd17b* and *Trim39* had no mice with bilateral phenotypes. However, these genes may be required for the ocular tear film, corneal epithelial integrity, or corneal wound healing. Therefore, these corneas may be less durable in response to an environmental stressor (such as mild trauma or bacterial seeding) and thus vulnerable to injury, infection, or fibrotic wound response, explaining how a germline deletion can lead to unilateral phenotypes. Furthermore, the limited IMPC ontology terms and thin nature of the mouse cornea limit precise identification of the location within the cornea where the observed CD occurs. This, along with the difference in corneal structure between mice and humans, impedes the direct correlation between mouse corneal phenotypes and clinical CDs. This indirect relationship could contribute to the identification of novel human cornea genes, as some mouse cornea genes may not play a role in the human cornea. Narrowing the focus to candidate LACD genes expressed in human cornea tissues derives greater clinical implication from this study. Future analysis should examine precise layers within the cornea so relationships can be more accurately linked to disrupted pathways.

## Conclusions

In this investigation, 14 genes associated with LACD were identified from a pool of 587 IMPC knockout mouse lines that went through late-adult phenotyping, 12 of which are only now implicated in corneal biology and 7 are found to be expressed in the human cornea. These genes may represent underappreciated biological processes important for the aging cornea. Further studies may prove them to be potential therapeutic targets for preventing or treating corneal diseases in aging human populations.

## Supplementary Material

Supplement 1

Supplement 2

Supplement 3

Supplement 4

Supplement 5

Supplement 6

Supplement 7

Supplement 8

Supplement 9

Supplement 10

Supplement 11
