# Supplementary material for: Systematic Ocular Phenotyping of Knockout Mouse Lines Identifies Genes Associated With Age-Related Corneal Dystrophies
Source: Invest Ophthalmol Vis Sci. 2025 May 5;66(5):7. doi: 10.1167/iovs.66.5.7 (PMC12060066; doi:10.1167/iovs.66.5.7)
Supplement: Supplement 1 [file iovs-66-5-7_s001.pdf]

Supplemental Figure 1

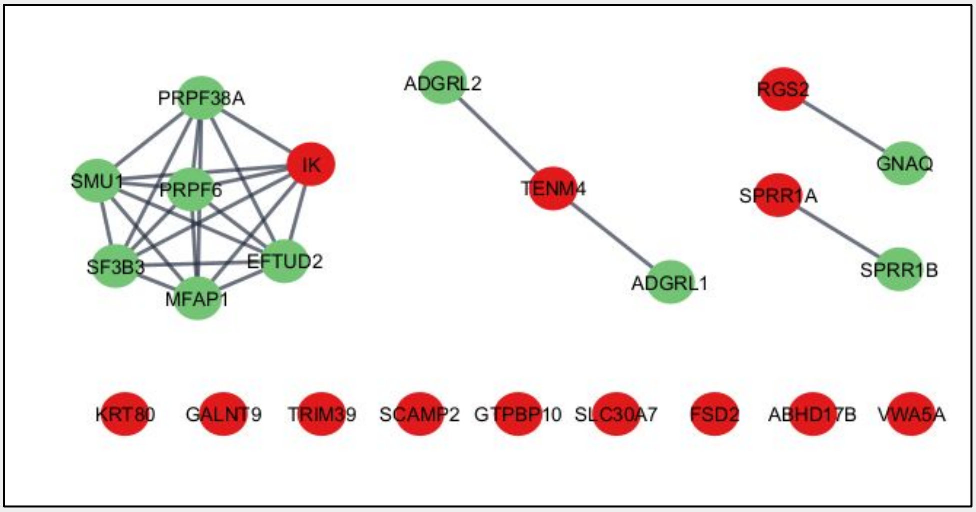

Supplemental Figure 1: Supplemental Figure 2: STRING protein-protein analysis between human ortholog proteins of 13 candidate LACD genes (red), and 10 additional interactor proteins determined by STRING (green). Gene Abca16 was omitted as it was not available in STRING. Analysis run with modified settings (Organism: Homo Sapiens; Network Type = full STRING network; Confidence cutoff 0.90; Additional interactors 10). Darker edges indicate stronger protein-protein interaction.
