# Supplementary material for: Systematic Ocular Phenotyping of Knockout Mouse Lines Identifies Genes Associated With Age-Related Corneal Dystrophies
Source: Invest Ophthalmol Vis Sci. 2025 May 5;66(5):7. doi: 10.1167/iovs.66.5.7 (PMC12060066; doi:10.1167/iovs.66.5.7)
Supplement: Supplement 2 [file iovs-66-5-7_s002.pdf]

Supplemental Figure 2

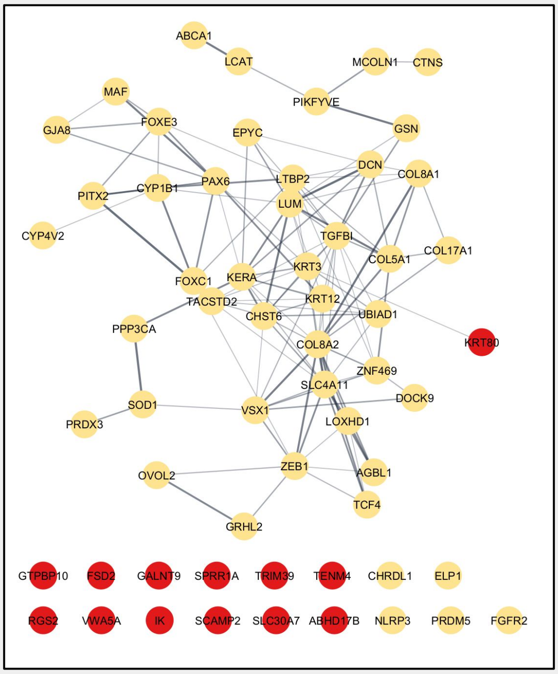

Supplemental Figure 2: STRING protein-protein analysis between human ortholog proteins of 13 candidate LACD genes (red) and 47 established human CD genes (gold). Candidate LACD gene *Abca16* and established human CD gene *MIR-184* were omitted from this analysis as they are not available in STRING. Analysis run with modified settings (Organism: Homo Sapiens; Network Type = full STRING network; Confidence cutoff 0.40). Darker edges indicate stronger protein-protein interaction.
