# Supplementary material for: Systematic Ocular Phenotyping of Knockout Mouse Lines Identifies Genes Associated With Age-Related Corneal Dystrophies
Source: Invest Ophthalmol Vis Sci. 2025 May 5;66(5):7. doi: 10.1167/iovs.66.5.7 (PMC12060066; doi:10.1167/iovs.66.5.7)
Supplement: Supplement 3 [file iovs-66-5-7_s003.pdf]

Supplemental Figure 3

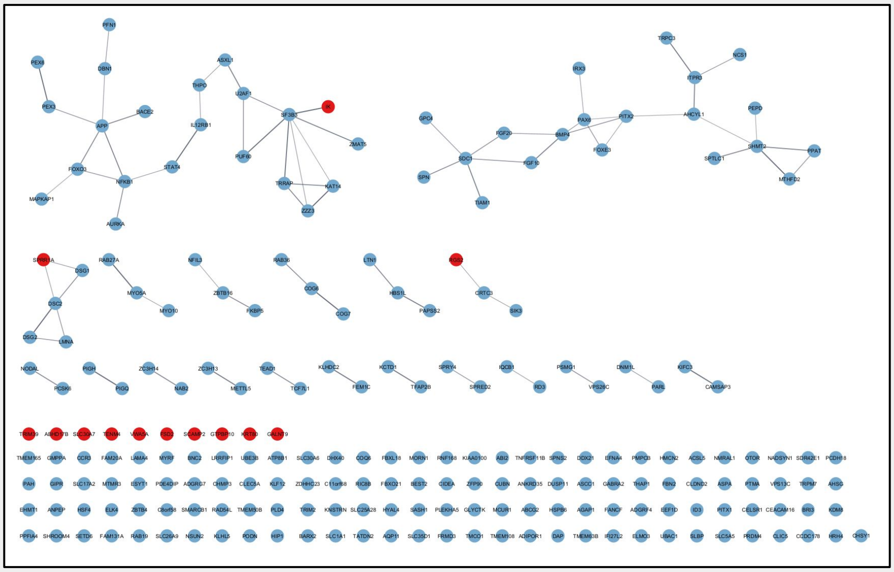

Supplemental Figure 3: STRING protein-protein analysis between 13 candidate LACD genes (red) and 205 early-stage CD genes (blue). Candidate LACD gene *Abca16* and 8 early-stage CD genes, Ahsa2, Gimap3, Lpcat2b, Mir-96, Ngp, Skic2, Str6la, and Zfp395 were omitted from this analysis as they are not available in STRING. Analysis run with modified settings (Organism: Homo Sapiens; Network Type = full STRING network; Confidence cutoff 0.50). Darker edges indicate stronger protein-protein interaction.
