# Supplementary material for: Systematic Ocular Phenotyping of Knockout Mouse Lines Identifies Genes Associated With Age-Related Corneal Dystrophies
Source: Invest Ophthalmol Vis Sci. 2025 May 5;66(5):7. doi: 10.1167/iovs.66.5.7 (PMC12060066; doi:10.1167/iovs.66.5.7)
Supplement: Supplement 4 [file iovs-66-5-7_s004.pdf]

Supplemental Figure 4

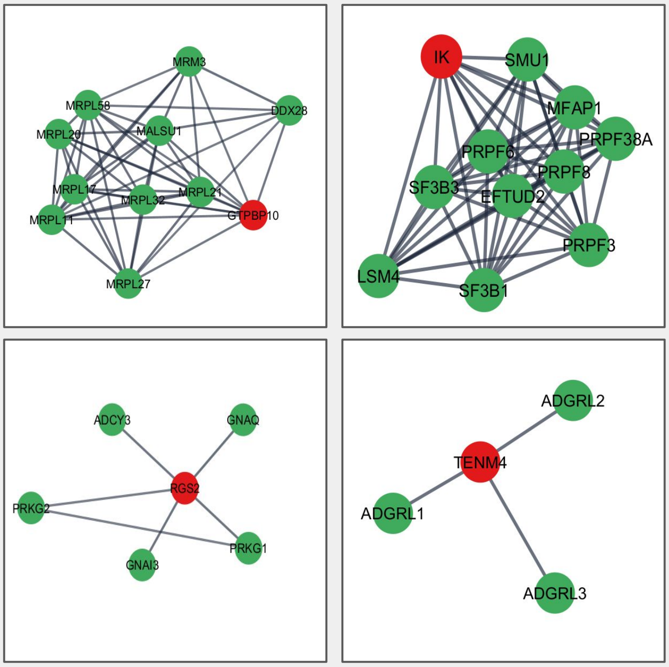

Supplemental Figure 4: STRING protein-protein analysis of LACD genes expressed in human corneal tissue (red) with additional interactor proteins (green). Top row: Gtpbp10, Ik. Bottom row: Rgs2, Tenm4. Expressed LACD candidate genes Scamp2, Slc30a7, and Vwa5a were omitted as no protein networks were established at the highest confidence level. Analysis run with modified settings (Organism: Homo Sapiens; Network Type = full STRING network; Confidence cutoff 0.90; Additional interactors 10). Darker edges indicate stronger protein-protein interaction.
