# Supplementary material for: Systematic Ocular Phenotyping of Knockout Mouse Lines Identifies Genes Associated With Age-Related Corneal Dystrophies
Source: Invest Ophthalmol Vis Sci. 2025 May 5;66(5):7. doi: 10.1167/iovs.66.5.7 (PMC12060066; doi:10.1167/iovs.66.5.7)
Supplement: Supplement 6 [file iovs-66-5-7_s006.pdf]

Supplemental Figure 6

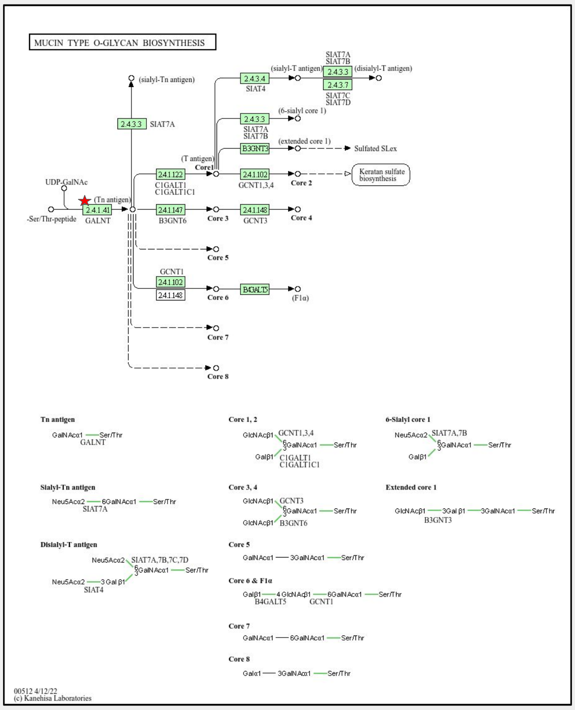

Supplemental Figure 6: Mucin type o-glycan biosynthesis KEGG pathway highlighting candidate gene *Galnt9* (red star).
