# Supplementary material for: Systematic Ocular Phenotyping of Knockout Mouse Lines Identifies Genes Associated With Age-Related Corneal Dystrophies
Source: Invest Ophthalmol Vis Sci. 2025 May 5;66(5):7. doi: 10.1167/iovs.66.5.7 (PMC12060066; doi:10.1167/iovs.66.5.7)
Supplement: Supplement 7 [file iovs-66-5-7_s007.pdf]

Supplemental Figure 7

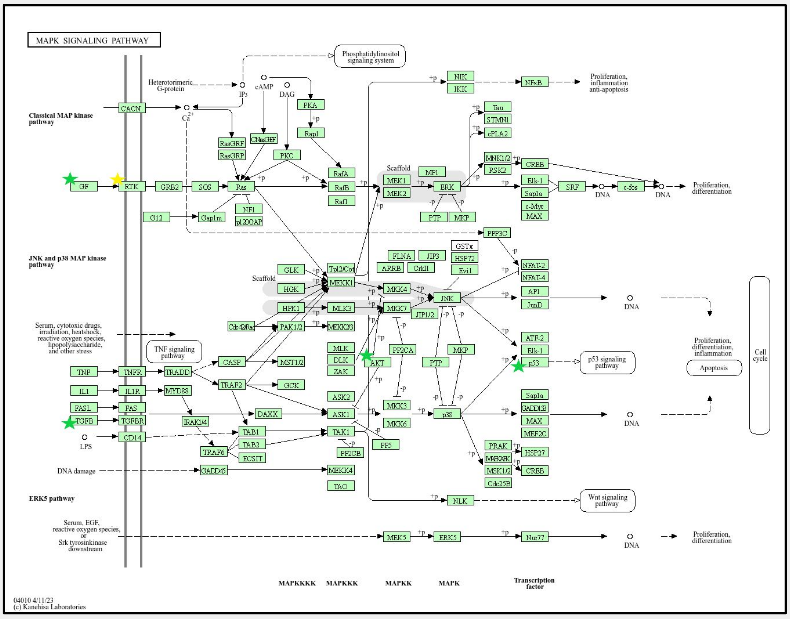

Supplemental Figure 7: MAPK signaling pathway highlighting established CD gene Fgfr2 (gold star) and STRING interactor genes Akt1, Tgfb1, Tp53, and Egf2 (green star).
