# Supplementary material for: Systematic Ocular Phenotyping of Knockout Mouse Lines Identifies Genes Associated With Age-Related Corneal Dystrophies
Source: Invest Ophthalmol Vis Sci. 2025 May 5;66(5):7. doi: 10.1167/iovs.66.5.7 (PMC12060066; doi:10.1167/iovs.66.5.7)
Supplement: Supplement 9 [file iovs-66-5-7_s009.pdf]

Supplemental Figure 9

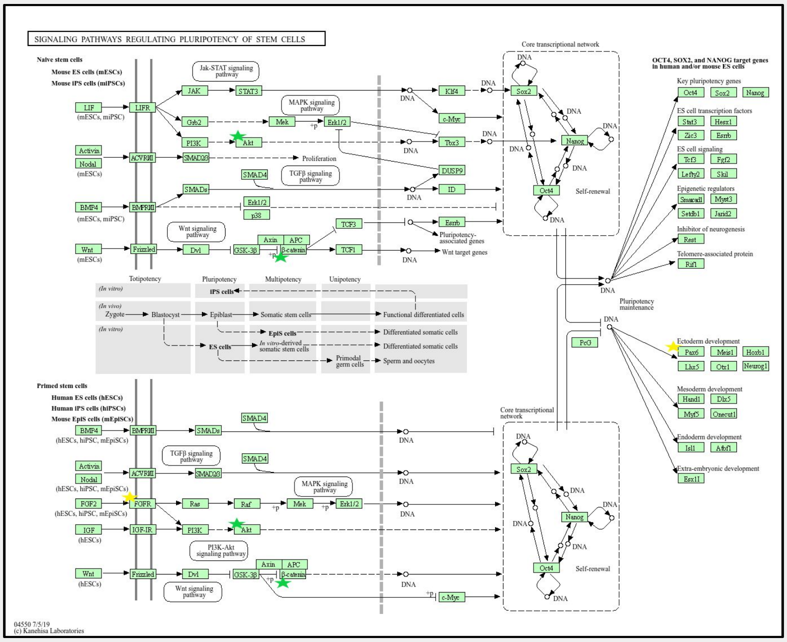

Supplemental Figure 9: Signaling Pathways Regulating Pluripotency of Stem Cells highlighting established CD genes Fgfr2 and Pax6 (gold star) and additional STRING interactor genes Akt1 and Ctnnb1 (green star).
