# Supplementary material for: Systematic Ocular Phenotyping of Knockout Mouse Lines Identifies Genes Associated With Age-Related Corneal Dystrophies
Source: Invest Ophthalmol Vis Sci. 2025 May 5;66(5):7. doi: 10.1167/iovs.66.5.7 (PMC12060066; doi:10.1167/iovs.66.5.7)
Supplement: Supplement 10 [file iovs-66-5-7_s010.pdf]

Supplemental Figure 10

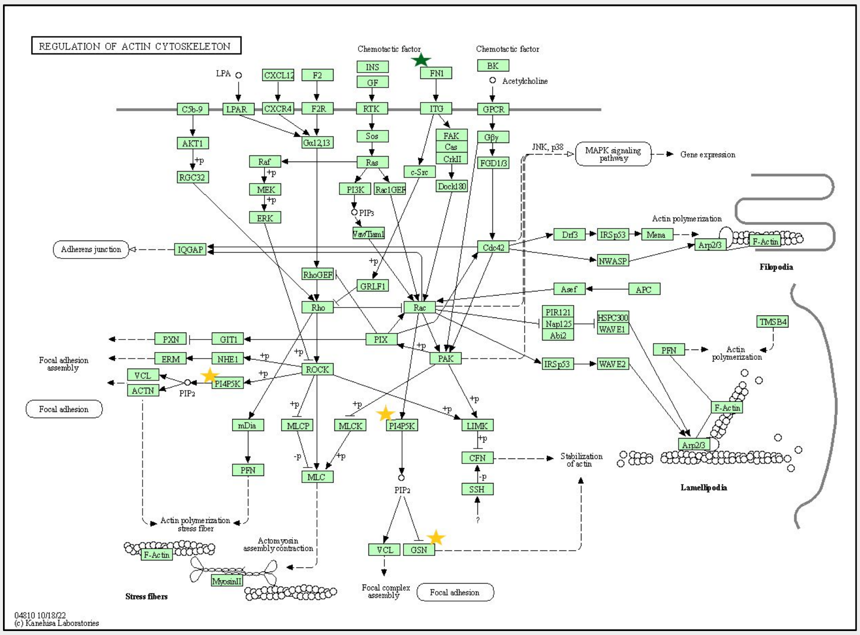

Supplemental Figure 10: Regulation of Actin Cytoskeleton pathway highlighting established human CD genes Pi4p5k, Gsn, and Fgfr2 (gold star), and additional STRING interactor genes Fn1 (green star).
