# Supplementary material for: Systematic Ocular Phenotyping of Knockout Mouse Lines Identifies Genes Associated With Age-Related Corneal Dystrophies
Source: Invest Ophthalmol Vis Sci. 2025 May 5;66(5):7. doi: 10.1167/iovs.66.5.7 (PMC12060066; doi:10.1167/iovs.66.5.7)
Supplement: Supplement 11 [file iovs-66-5-7_s011.pdf]

Supplemental Figure 11

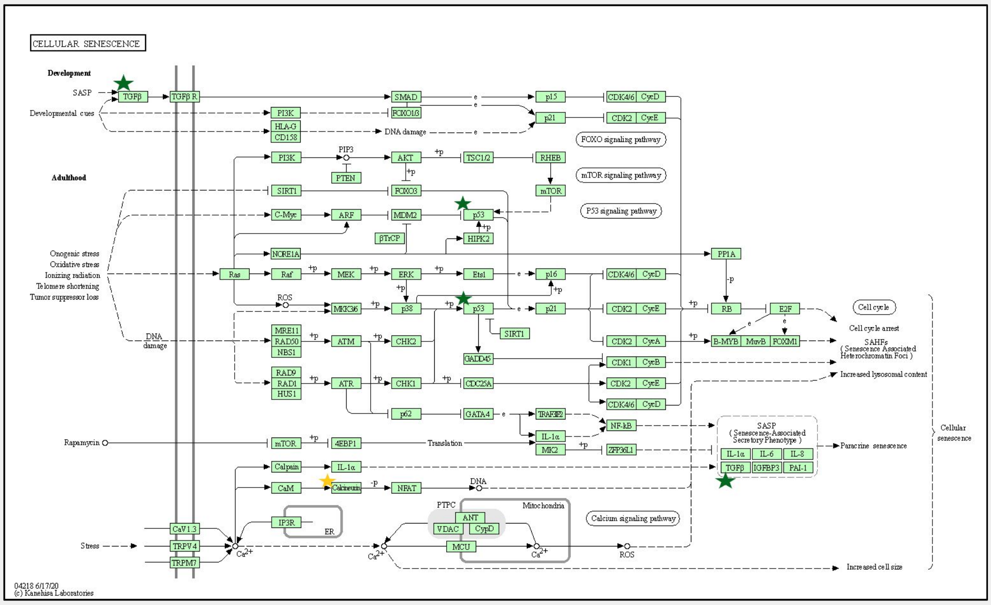

Supplemental Figure 11: Cellular Senescence pathway highlighting established human CD protein Cna1 (gold star) and additional STRING interactor gene Tp53 (green star).
